# Endocannabinoid metabolism inhibition has no effect on spontaneous fear recovery or extinction resistance in Lister hooded rats

**DOI:** 10.3389/fphar.2022.1082760

**Published:** 2022-12-15

**Authors:** William G. Warren, Eleni P. Papagianni, Ed Hale, Rebecca A. Brociek, Helen J. Cassaday, Carl W. Stevenson

**Affiliations:** ^1^ School of Biosciences, Sutton Bonington Campus, University of Nottingham, Loughborough, United Kingdom; ^2^ School of Veterinary Medicine and Science, University of Nottingham, Sutton Bonington Campus, Loughborough, United Kingdom; ^3^ School of Psychology, University Park, University of Nottingham, Nottingham, United Kingdom

**Keywords:** 2-arachidonoylglycerol, anandamide, anxiety, cannabinoid, fatty acid amide hydrolase, immediate extinction deficit, monoacylglycerol lipase, propranolol

## Abstract

Endocannabinoid transmission is emerging as a target for treating anxiety-related disorders, given its regulation of fear extinction. Boosting anandamide levels via inhibition of its metabolism by fatty acid amide hydrolase (FAAH) can enhance extinction, whereas inhibiting monoacylglycerol lipase (MAGL) to elevate 2-arachidonoylglycerol levels can impair extinction. However, whether endocannabinoids regulate fear relapse over time or extinction resistance remains unclear. In two experiments using auditory fear conditioned rats, we examined the effects of the FAAH inhibitor URB597 and the MAGL inhibitor JZL184 administered systemically on 1) spontaneous fear recovery after delayed extinction, and 2) extinction resistance resulting from immediate extinction [the immediate extinction deficit (IED)]. In Experiment 1, URB597 or JZL184 was given immediately after delayed extinction occurring 24 h after conditioning. Extinction recall and spontaneous fear recovery were tested drug-free 1 and 21 days later, respectively. We found no effects of either drug on extinction recall or spontaneous fear recovery. In Experiment 2, URB597 or JZL184 was given before immediate extinction occurring 30 min after conditioning and extinction recall was tested drug-free the next day. We also examined the effects of propranolol, a beta-adrenoceptor antagonist that can rescue the IED, as a positive control. JZL184 enhanced fear expression and impaired extinction learning but we found no lasting effects of URB597 or JZL184 on cued extinction recall. Propranolol reduced fear expression but, unexpectedly, had no enduring effect on extinction recall. The results are discussed in relation to various methodological differences between previous studies examining endocannabinoid and adrenergic regulation of fear extinction.

## Introduction

Anxiety-related disorders are associated with a significant prevalence and socioeconomic burden, given their inadequate treatment using currently available pharmacological and psychological therapies. Medications can lack full efficacy and have adverse side effects, while the benefits of psychological treatments are often temporary or limited outside of the therapeutic context. These issues can lead to symptom relapse over time, even after initially successful treatment, which highlights the drawbacks of these therapies (Di Luca et al., 2011; Baldwin et al., 2014; Craske et al., 2017). Various anxiety-related disorders are characterized by aberrant fear-related memories and deficiencies in their suppression through extinction, a form of inhibitory learning that diminishes learned fear expression. This deficit in extinction may contribute to certain limitations of psychological treatments since extinction forms the theoretical basis for exposure-based therapy (Michael et al., 2007; Milad et al., 2009, 2013; Kindt, 2014). To that end, a promising line of research is examining the use of pharmaceutics as adjuncts to strengthen extinction, with the aim of achieving lasting suppression of learned fear and symptom relapse (Karpova et al., 2011; Haaker et al., 2013; Singewald et al., 2015).

Endocannabinoid transmission has emerged as one such target for treating anxiety-related disorders due, in part, to the growing evidence for its role in regulating fear extinction (Lisboa et al., 2019; Papagianni and Stevenson, 2019; Mizuno and Matsuda, 2021; Warren et al., 2022a). Endocannabinoid signalling is mediated primarily by anandamide (AEA) and 2-arachidonoylglyecerol (2-AG) acting at cannabinoid receptor types 1 and 2 (CB1R and CB2R), along with other targets (e.g. transient receptor potential vanilloid 1 (TRPV1) channel) (Ligresti et al., 2016). Synaptic levels of AEA and 2-AG are controlled by transporter-mediated reuptake and degradative enzymes that mediate their metabolism. Fatty acid amide hydrolase (FAAH) and monoacylglycerol lipase (MAGL) preferentially break down AEA and 2-AG, respectively (Cravatt et al., 1996; Dinh et al., 2002). CB1R antagonism impairs and CB1R agonism enhances fear extinction (Lisboa et al., 2019; Mizuno and Matsuda, 2021; Warren et al., 2022a). However, the psychotropic side effects of direct CB1R agonists likely precludes their use as anxiolytics. In contrast, indirect CB1R agonism via elevated endocannabinoid levels resulting from inhibition of their metabolism is a more feasible therapeutic target. FAAH inhibition has been shown to enhance fear extinction in a CB1R-dependent manner (Laricchiuta et al., 2013; Gunduz-Cinar et al., 2013; Llorente-Berzal et al., 2015; Segev et al., 2018; Morena et al., 2018), whereas MAGL inhibition can impair fear extinction (Llorente-Berzal et al., 2015; Hartley et al., 2016; Mizuno et al., 2022). These findings suggest opposing roles for AEA and 2-AG in fear extinction, although whether they also regulate fear relapse over time and extinction resistance remains poorly understood.

Here we used rats subjected to auditory fear conditioning to investigate the effects of the FAAH inhibitor URB597 and the MAGL inhibitor JZL184 on fear relapse over time and extinction resistance. After successful extinction, fear can return with the passage of time through the process of spontaneous fear recovery (Bouton et al., 2006), which models fear relapse over time. Compared to delayed extinction occurring 24 h after fear conditioning, immediate extinction (<6 h) after more recent conditioning results in impaired extinction. This immediate extinction deficit (IED) models extinction resistance (Maren, 2022). In Experiment 1, we examined the effects of different drug doses given after delayed extinction on extinction recall and later spontaneous fear recovery. In Experiment 2, we examined the effects of drug given before immediate extinction on extinction and its recall. Drug effects in no extinction controls were examined to determine their potential dependence on extinction and effects on fear memory consolidation. The IED is thought to result from stress induced by recent fear conditioning. This is supported by evidence indicating that blocking receptor signalling mediated by stress mediators, such as noradrenaline, can rescue the IED (Fitzgerald et al., 2015; Giustino et al., 2017, 2020). Therefore, we also examined the effects of the beta-adrenoceptor antagonist propranolol on immediate extinction as a positive control.

## Materials and methods

### Animals

Adult male Lister hooded rats (Charles River or Envigo, United Kingdom) that weighed 200–300 g upon arrival 10 days before the start of experiments were used in this study. Rats were group housed (4/cage) in individually ventilated cages on a 12 h light/dark cycle (lights on at 8:00) with food and water available *ad libitum*. Upon completion of the experiments rats were humanely culled with a rising concentration of CO_2_. All experimental procedures were conducted with prior institutional ethical approval and in accordance with the Animals (Scientific Procedures) Act 1986, United Kingdom (Home Office Project Licences 30/3,230 and P6DA59444).

### Drug administration

URB597 and JZL184 (Sigma-Aldrich, United Kingdom) were suspended in a vehicle of 5% polyethylene glycol, 5% Tween 80, and sterile saline. Propranolol (Fisher Scientific, United Kingdom) was dissolved in sterile saline. Drug solutions were made up on the day of use. In Experiment 1, URB597 (0.1, 0.3, and 1 mg/kg), JZL184 (1, 3, and 10 mg/kg), or vehicle was injected (i.p, 1 ml/kg) at dose ranges used in previous fear extinction studies (Laricchiuta et al., 2013; Llorente-Berzal et al., 2015; Hartley et al., 2016; Segev et al., 2018; Morena et al., 2018, 2021; Mizuno et al., 2022). In Experiment 2, one dose of URB597, JZL184, propranolol, or their respective vehicles were used. The doses used for URB597 (1 mg/kg) and JZL184 (10 mg/kg) were the highest doses used in Experiment 1, while the dose used for propranolol (10 mg/kg) was based on a previous study showing that it rescues the IED (Fitzgerald et al., 2015).

### Behavioural testing

The behavioural testing procedures used were based on our recent study on delayed and immediate extinction (Papagianni et al., 2022). Four behavioural testing chambers were used, which have been described elsewhere (Stevenson et al., 2009). Presentations of tone and footshock were controlled via a PC running MED-PC V software (Med Associates, US). All behavioural testing occurred during the rats’ light cycle and behaviour was recorded for later data analysis (see below).

**FIGURE 1:**
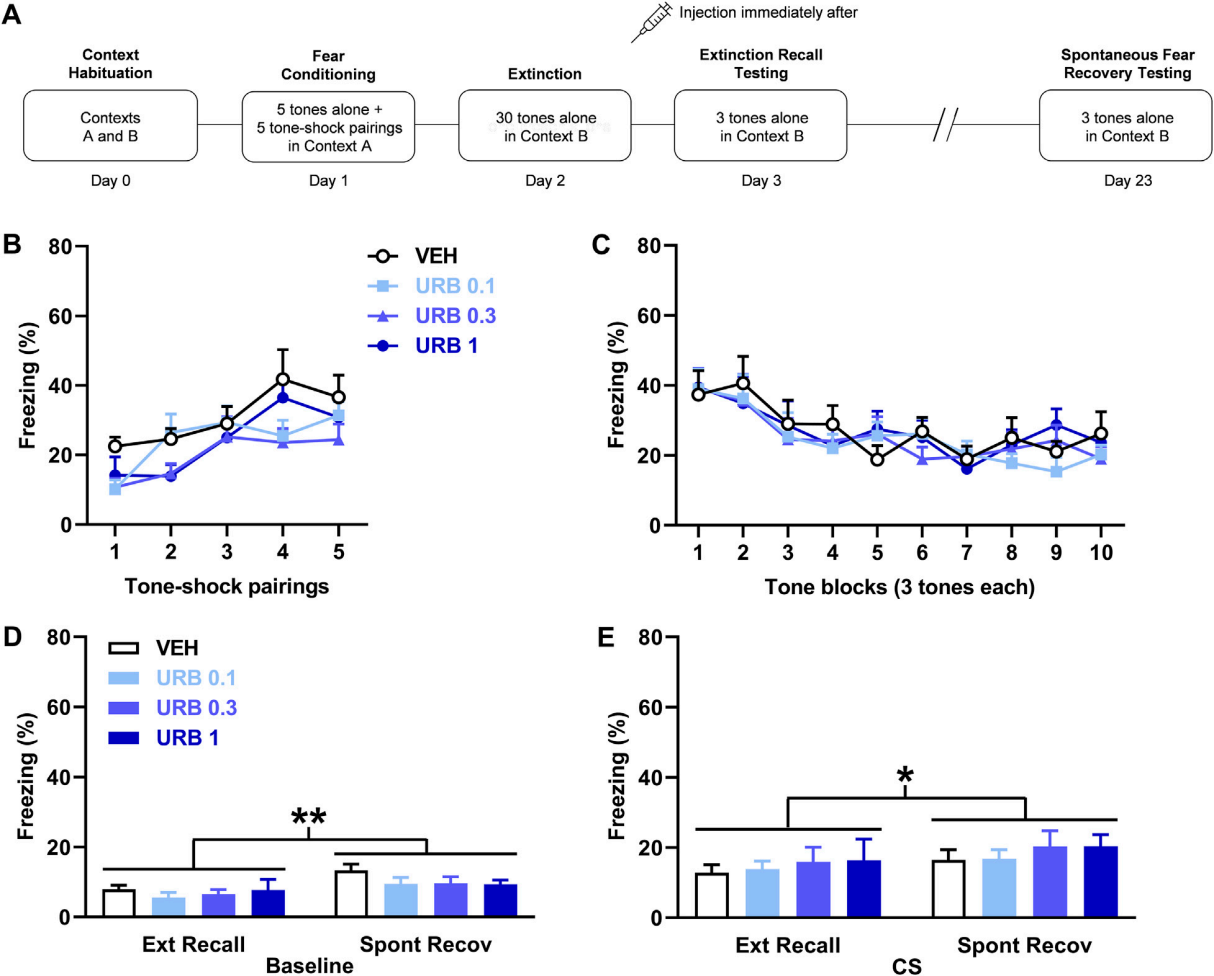
URB597 (URB) given after delayed extinction has no effect on extinction recall or spontaneous fear recovery. **(A)** Experimental design showing the behavioural testing and drug administration procedures used (*n* = 12/group). **(B,C)** There were no differences in freezing in response to tone-shock pairings during fear conditioning **(B)** or tones during extinction **(C)** between the groups to receive vehicle (VEH) or the different URB doses (0.1 mg/kg: URB 0.1; 0.3 mg/kg: URB 0.3; 1 mg/kg: URB 1) after delayed extinction. **(D)** Freezing before tone presentations (Baseline) was increased during spontaneous fear recovery (Spont Recov), compared to extinction recall (Ext Recall), testing (**p < 0.01) but URB had no effect on freezing. **(E)** Freezing in response to the tones (CS) was increased during Spont Recov, compared to Ext Recall, testing (*p < 0.05) but URB had no effect on freezing.

**FIGURE 2:**
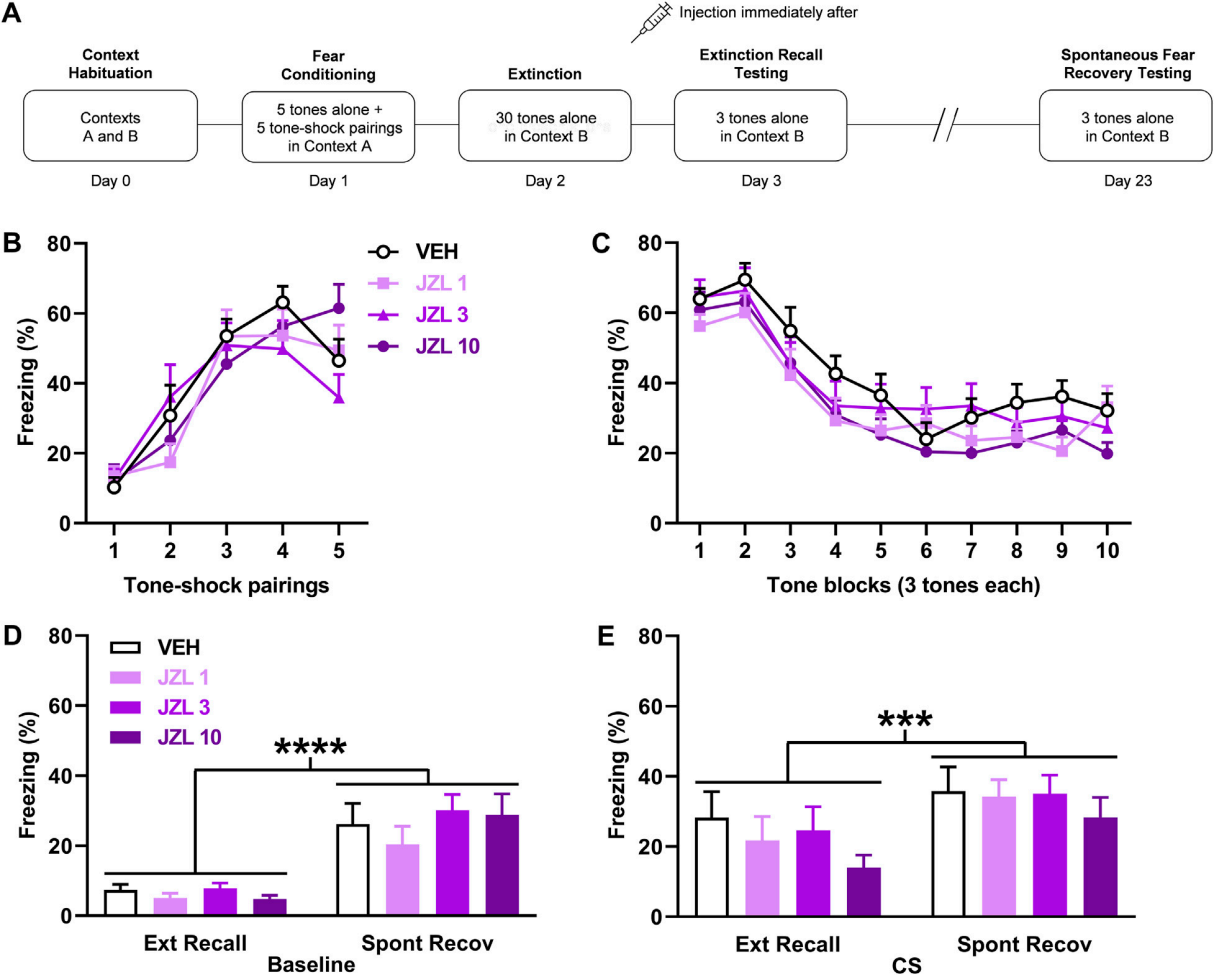
JZL184 (JZL) given after delayed extinction has no effect on extinction recall or spontaneous fear recovery. **(A)** Experimental design showing the behavioural testing and drug administration procedures used (*n* = 12/group). **(B,C)** There were no differences in freezing in response to tone-shock pairings during fear conditioning **(B)** or tones during extinction **(C)** between the groups to receive vehicle (VEH) or the different JZL doses (1 mg/kg: JZL 1; 3 mg/kg: JZL 3; 10 mg/kg: JZL 10) after delayed extinction. **(D)** Freezing before tone presentations (Baseline) was increased during spontaneous fear recovery (Spont Recov), compared to extinction recall (Ext Recall), testing (****p < 0.0001) but JZL had no effect on freezing. **(E)** Freezing in response to the tones (CS) was increased during Spont Recov, compared to Ext Recall, testing (***p < 0.001) but JZL had no effect on freezing.

In Experiment 1, we investigated the effects of URB597 (Figure 1A) or JZL184 (Figure 2A) given immediately after delayed extinction on extinction recall and later spontaneous fear recovery. Rats were randomly allocated to receive one of the drug doses or vehicle (n = 12/group). On Day 0, rats were habituated to two distinct contexts (A and B; 10 min each). On Day 1, rats underwent auditory fear conditioning in context A, consisting of five tones presented alone (30 s, 4 kHz, 80 dB, 2 min inter-trial interval (ITI)) followed by five pairings of the tone with footshock (0.5 s, 0.4 mA, ending at tone offset). On Day 2, rats underwent extinction in context B, which consisted of 30 tones presented alone (30 s ITI). Immediately after extinction rats were injected with drug or vehicle. On Day 3, rats underwent extinction recall testing drug-free in context B, which consisted of three tones presented alone (30 s ITI). On Day 23, rats underwent spontaneous fear recovery testing drug-free in context B as for extinction recall testing.

**FIGURE 3:**
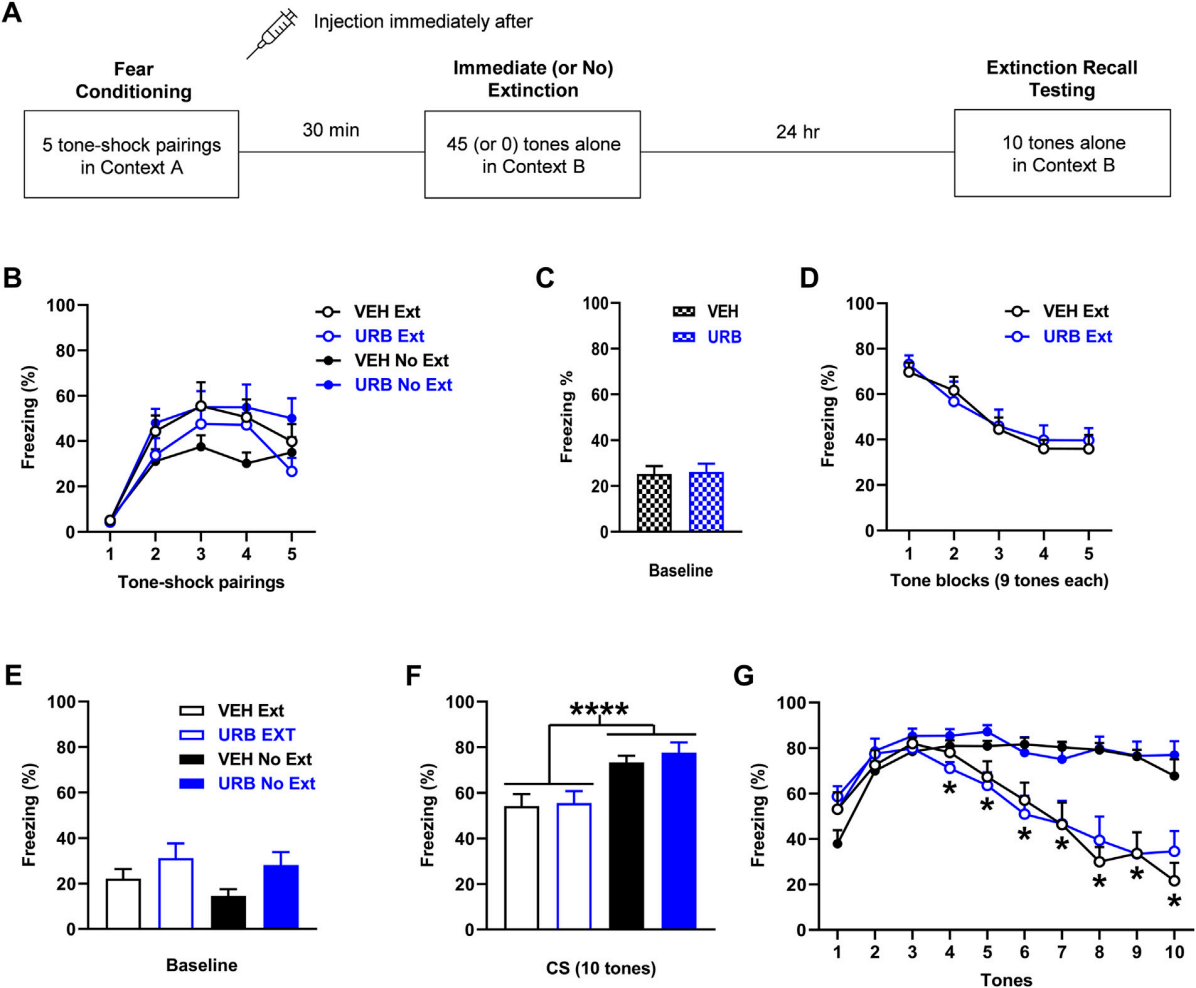
URB597 (URB; 1 mg/kg) given before immediate extinction has no effect on fear expression or extinction. **(A)** Experimental design showing the behavioural testing and drug administration procedures used (*n* = 10/group). **(B)** There were no differences in freezing in response to tone-shock pairings during fear conditioning between the groups to receive vehicle (VEH) or URB before immediate extinction (VEH Ext, URB Ext). However, freezing was increased in the group to receive URB before no extinction (URB No Ext), compared to the group to receive VEH before no extinction (VEH No Ext). **(C)** There were no differences in freezing before tone presentations (Baseline) during immediate (or no) extinction between VEH and URB. **(D)** URB had no effect on tone-induced freezing during immediate extinction. **(E)** Compared to VEH, URB resulted in increased freezing before tone presentations (Baseline) during extinction recall testing (*p* < 0.05). **(F)** Tone-induced freezing across extinction recall testing was decreased with Ext, compared to No Ext (****p < 0.0001), but URB resulted in no effect on freezing. **(G)** Freezing in response to each tone during extinction recall testing was decreased with Ext, compared to No Ext, at tones 4–10 (*p < 0.05) but freezing was unaffected by URB.

**FIGURE 4:**
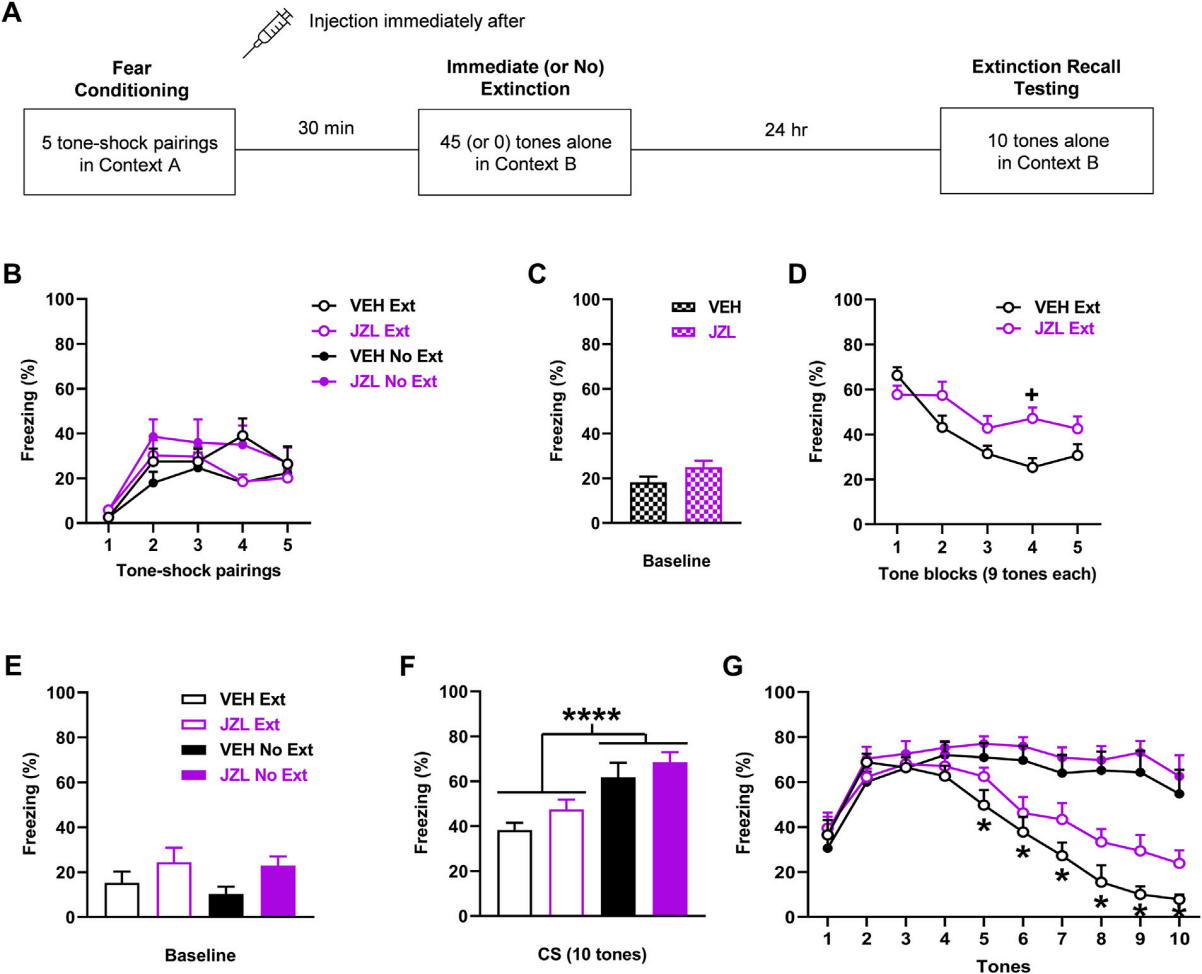
JZL184 (JZL; 10 mg/kg) given before immediate extinction enhances fear expression and impairs extinction learning without affecting extinction encoding. **(A)** Experimental design showing the behavioural testing and drug administration procedures used (n = 10/group). **(B)** There were no differences in freezing in response to tone-shock pairings during fear conditioning between the groups to receive vehicle (VEH) or JZL before immediate extinction (VEH Ext, JZL Ext) or no extinction (VEH No Ext, JZL No Ext). **(C)** There were no differences in freezing before tone presentations (Baseline) during immediate (or no) extinction between VEH and JZL. **(D)** Compared to VEH, JZL increased freezing during immediate extinction (^+^ *p* < 0.05) and impaired extinction learning. **(E)** JZL resulted in increased freezing before tone presentations (Baseline) during extinction recall testing, compared to VEH (*p* < 0.05). **(F)** Tone-induced freezing across extinction recall testing was decreased with Ext, compared to No Ext (*p < 0.05), but JZL resulted in no effect on freezing. **(G)** Freezing in response to each tone during extinction recall testing was decreased with Ext, compared to No Ext, at tones 5–10 (*p < 0.05) but freezing was unaffected by JZL.

**FIGURE 5:**
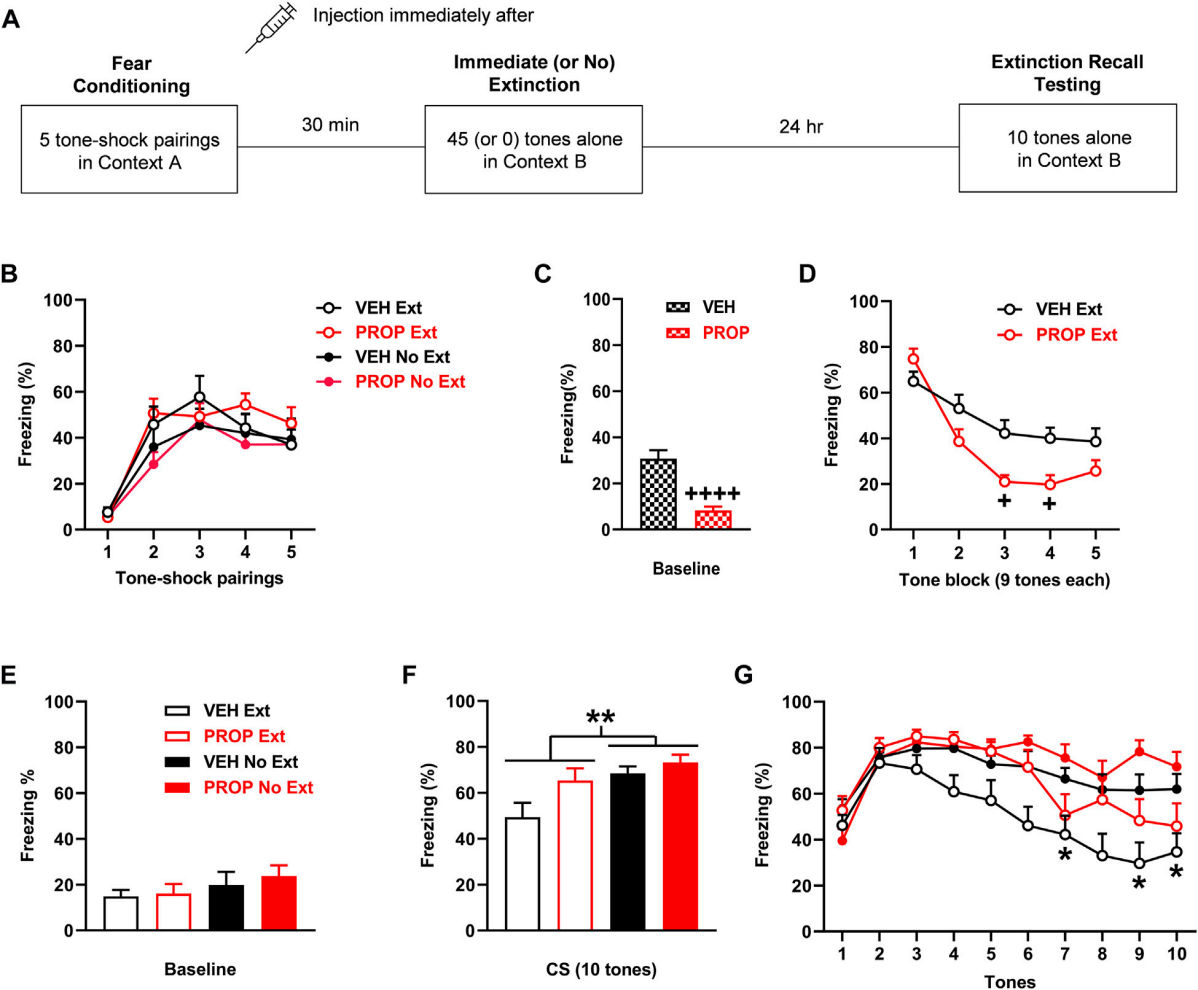
Propranolol (PROP; 10 mg/kg) given before immediate extinction reduces fear expression without affecting extinction encoding. **(A)** Experimental design showing the behavioural testing and drug administration procedures used (*n* = 10/group). **(B)** There were no differences in freezing in response to tone-shock pairings during fear conditioning between the groups to receive vehicle (VEH) or PROP before immediate extinction (VEH Ext, PROP Ext) or no extinction (VEH No Ext, PROP No Ext). **(C)** Compared to VEH, PROP decreased freezing before tone presentations (Baseline) during immediate (or no) extinction (^++++^ *p* < 0.0001). **(D)** PROP decreased tone-induced freezing during immediate extinction, compared to VEH (^+^ *p* < 0.05). **(E)** PROP resulted in no effect on freezing before tone presentations (Baseline) during extinction recall testing. **(F)** Tone-induced freezing across extinction recall testing was increased with PROP in comparison to VEH (*p* < 0.05) and decreased with Ext, compared to No Ext (**p < 0.01). **(G)** Freezing in response to each tone during extinction recall testing was decreased with Ext, compared to No Ext, at tones 7 and 9–10 (*p < 0.05) but freezing was unaffected by PROP.

In Experiment 2, we examined the effects of URB597 (Figure 3A) or JZL184 (Figure 4A) given before immediate extinction on extinction recall to determine if either drug rescues the IED. We also examined the effects of propranolol (Figure 5A) as a positive control since this drug has been shown to rescue the IED (Fitzgerald et al., 2015). Rats were randomly allocated to the four following groups: drug immediate extinction, vehicle immediate extinction, drug no extinction, vehicle no extinction (*n* = 10/group). All rats underwent auditory fear conditioning, consisting of five tones (as above) paired with footshock (1 s, 0.5 mA) in context A. Stronger footshocks were used than in Experiment 1 since weaker fear conditioning fails to induce the IED (Maren and Chang, 2006) and we have demonstrated the IED using these parameters (Papagianni et al., 2022). Immediately after the end of fear conditioning rats received drug or vehicle treatment and were then returned to their home cage. Immediate extinction occurred 30 min after conditioning and consisted of 45 tones presented alone (30 s ITI) in context B. No extinction also occurred 30 min after conditioning but the rats were placed in context B for the same duration without any tone presentations. All rats underwent extinction recall testing 24 h after immediate or no extinction, which consisted of 10 tones presented alone (30 s ITI) in context B.

### Data analysis

Freezing, defined as the absence of movement except for that related to respiration, in response to tone-shock pairings during fear conditioning and tone presentations during extinction, extinction recall testing (Experiments 1-2), and spontaneous fear recovery testing (Experiment 1) was quantified as an index of learned fear. Automated freezing scoring with VideoTrack software (ViewPoint, France) was used as we have previously described (Stubbendorff et al., 2019). The cumulative duration of freezing during each tone was calculated and expressed as a percentage of tone duration. Baseline fear expression at the start of extinction (Experiment 2), extinction recall testing (Experiments 1, 2), and spontaneous fear recovery testing (Experiment 1) was inferred from freezing levels during the 2 min period before tone presentations and quantified as above.

In Experiment 1, the mean percentage of freezing in response to three consecutive tones during delayed extinction was calculated and used in the analysis. The mean percentage of freezing in response to the three tones during extinction recall and spontaneous fear recovery testing was also calculated to use in the analysis. Differences in freezing in response to tone-shock pairings during fear conditioning were analyzed using two-way analysis of variance (ANOVA), with dose and trial as between- and within-subject factors, respectively. In the JZL184 experiment, the fear conditioning data from one animal in each group was lost due to equipment failure, resulting in n = 11/group (all other datasets included *n* = 12/group). Differences in tone-induced freezing during extinction were also analyzed using two-way ANOVA, with dose and trial block as between- and within-subject factors, respectively. Differences in baseline freezing during extinction recall and spontaneous fear recovery testing were analyzed using two-way ANOVA, with dose and session as between- and within-subjects factors, respectively. Differences in tone-induced freezing during extinction recall and spontaneous fear recovery testing were analyzed in the same way.

In Experiment 2, the mean percentage of freezing in response to nine consecutive tones during immediate extinction was calculated and used in the analysis. Similarly, the mean percentage of freezing in response to the 10 tones during extinction recall was calculated to use in the analysis. Differences in freezing in response to tone-shock pairings during fear conditioning were analyzed using three-way ANOVA, with treatment (drug vs. vehicle) and training (extinction vs. no extinction) as between-subject factors and trial as a within-subject factor. Baseline freezing data before extinction from the two drug (extinction and no extinction) and two vehicle (extinction and no extinction) groups were combined. Differences in baseline freezing before immediate extinction were analyzed using two-tailed unpaired t-tests. Differences in tone-induced freezing during immediate extinction were analyzed using two-way ANOVA as above. Differences in baseline freezing during extinction recall testing were analyzed using two-way ANOVA, with treatment and training as between-subjects factors. Differences in tone-induced freezing during extinction recall testing were analyzed in two ways. Differences in mean freezing across the 10 tones were analyzed using two-way ANOVA, with treatment and training as between-subjects factors. Differences in freezing in response to each tone were also analyzed using three-way ANOVA, with treatment and training as between-subject factors and trial as a within-subject factor.

All data are presented as the mean +standard error of the mean. The Geisser-Greenhouse correction was applied to all repeated measures ANOVAs. Post-hoc comparisons were conducted using the Tukey’s or Sidak’s tests where indicated. The level of significance for all comparisons was *p* < 0.05.

## Results

### Experiment 1

In Experiment 1 we determined the effects of different doses of URB597 or JZL184 on the consolidation of delayed extinction and later spontaneous fear recovery. Drug (or vehicle) was given immediately after delayed extinction, with extinction recall and spontaneous fear recovery tested drug-free 1 and 21 days later, respectively.

### URB597 given after delayed extinction has no effect on extinction recall or spontaneous fear recovery

The effects of URB597 (*n* = 12/group) given after delayed extinction on extinction recall and spontaneous fear recovery are shown in Figure 1. There were no differences in freezing in response to tone-shock pairings during fear conditioning between the groups (Figure 1B). Two-way ANOVA showed no main effect of dose (F _(3, 44)_ = 1.79, *p* = 0.16) or dose × trial interaction (F _(12, 176)_ = 1.25, *p* = 0.26). Similarly, there were no differences in freezing between the groups in response to tone presentations during delayed extinction (Figure 1C). Again, two-way ANOVA showed no main effect of dose (F _(3, 44)_ = 0.13, *p* = 0.94) or dose x trial block interaction (F _(27, 396)_ = 0.62, *p* = 0.93). Freezing before tone presentations was increased during spontaneous fear recovery, compared to extinction recall, testing but URB597 had no effect on freezing in either session (Figure 1D). Two-way ANOVA showed a significant main effect of session (F _(1, 44)_ = 11.05, *p* = 0.0018) but no main effect of dose (F _(3, 44)_ = 0.85, *p* = 0.47) or dose × session interaction (F _(3, 44)_ = 0.60, *p* = 0.62). Tone-induced freezing was also increased during spontaneous fear recovery, compared to extinction recall, testing but, again, URB597 had no effect on freezing in either session (Figure 1E). Two-way ANOVA showed a significant main effect of session (F _(1, 44)_ = 6.37, *p* = 0.015) but no main effect of dose (F _(3, 44)_ = 0.31, *p* = 0.82) or dose × session interaction (F _(3, 44)_ = 0.039, *p* = 0.99). This indicates that the spontaneous recovery of baseline and tone-induced fear occurred but were unaffected by prior URB597 treatment.

### JZL184 given after delayed extinction has no effect on extinction recall or spontaneous fear recovery

The effects of JZL184 (*n* = 12/group) given after delayed extinction on extinction recall and spontaneous fear recovery are shown in Figure 2. Freezing in response to tone-shock pairings during fear conditioning did not differ between the groups (Figure 2B). Two-way ANOVA showed no main effect of dose (F _(3, 40)_ = 0.27, *p* = 0.85) or dose × trial interaction (F _(12, 160)_ = 1.47, *p* = 0.14). There were also no differences in freezing between the groups in response to tone presentations during delayed extinction (Figure 2C). Two-way ANOVA showed no main effect of dose (F _(3, 44)_ = 1.64, *p* = 0.19) or dose x trial block interaction (F _(27, 396)_ = 0.71, *p* = 0.85). Freezing before tone presentations was increased during spontaneous fear recovery, compared to extinction recall, testing but JZL184 had no effect on freezing in either session (Figure 2D). Two-way ANOVA showed a significant main effect of session (F _(1, 44)_ = 61.99, *p* < 0.0001) but no main effect of dose (F _(3, 44)_ = 0.73, *p* = 0.54) or dose × session interaction (F _(3, 44)_ = 0.56, *p* = 0.64). Similarly, tone-induced freezing was increased during spontaneous fear recovery, compared to extinction recall, testing but JZL184 had no effect on freezing in either session (Figure 2E). Two-way ANOVA showed a significant main effect of session (F _(1, 44)_ = 12.65, *p* = 0.0009) but no main effect of dose (F _(3, 44)_ = 0.83, *p* = 0.48) or dose × session interaction (F _(3, 44)_ = 0.21, *p* = 0.89). This indicates that the spontaneous recovery of baseline and tone-induced fear occurred but were unaffected by prior JZL184 treatment.

### Experiment 2

In Experiment 2, we determined the effects of URB597 or JZL184 on learned fear expression and immediate extinction after recent fear conditioning. Drug (or vehicle) was given before immediate extinction and extinction recall was tested drug-free the next day. Compared to Experiment 1, we used stronger shock parameters during fear conditioning in order to induce the IED (Maren and Chang, 2006). No extinction controls were also included to examine potential drug effects on fear memory consolidation and their potential dependence on extinction. Although we have recently validated this IED procedure (Papagianni et al., 2022), we used weaker shock parameters than those used by others to demonstrate the IED. These previous studies have provided evidence that the IED involves heightened noradrenaline transmission resulting from the stress associated with recent fear conditioning, given that the IED was rescued by propranolol (Fitzgerald et al., 2015; Giustino et al., 2017, 2020). Therefore, we also examined the effects of propranolol on immediate extinction as a positive control and in an attempt to replicate its effect on the IED using our weaker shock parameters.

### URB597 given before immediate extinction has no effect on fear expression or extinction

The effects of URB597 (*n* = 10/group) given before immediate extinction on learned fear expression, extinction, and extinction recall are shown in Figure 3. Freezing in response to tone-shock pairings during fear conditioning differed between the two no extinction groups (Figure 3B). Three-way ANOVA showed no main effects of treatment (F _(1, 36)_ = 0.79, *p* = 0.38) or training (F _(1, 36)_ = 0.0057, *p* = 0.94) but there was a significant treatment × training interaction (F _(1, 36)_ = 6.67, *p* = 0.014). Post-hoc analysis showed that freezing across the tone-shock pairings was increased in the URB597 no extinction group, compared to the vehicle no extinction group (*p* < 0.05). Despite this difference in freezing during conditioning, there were no differences in freezing before tone presentations during immediate (or no) extinction between the vehicle and URB597 groups (t _(38)_ = 0.18, *p* = 0.86), indicating a lack of effect of URB597 on baseline fear expression during immediate extinction (Figure 3C). Similarly, URB597 had no effect on tone-induced freezing during immediate extinction (Figure 3D). Two-way ANOVA showed no main effect of treatment (F _(1, 18)_ = 0.048, *p* = 0.83) or treatment x trial block interaction (F _(4, 72)_ = 0.46, *p* = 0.77), indicating a lack of effect of URB597 on cued fear expression or extinction learning. Freezing before tone presentations during extinction recall testing was increased with URB597, compared to vehicle (Figure 3E). Two-way ANOVA showed a significant main effect of treatment (F _(1, 36)_ = 5.06, *p* = 0.031) but no treatment × training interaction (F _(1, 36)_ = 0.20, *p* = 0.65), indicating that URB597 resulted in increased baseline fear expression at extinction recall. Tone-induced freezing during extinction recall testing based on mean freezing across the 10 tones was decreased with immediate, compared to no, extinction but this was unaffected by URB597 (Figure 3F). Two-way ANOVA showed a significant main effect of training (F _(1, 36)_ = 20.31, *p* < 0.0001) but no main effect of treatment (F _(1, 36)_ = 0.38, *p* = 0.54) or treatment × training interaction (F _(1, 36)_ = 0.10, *p* = 0.75). This was confirmed by examining freezing in response to each tone (Figure 3G). Three-way ANOVA showed a significant main effect of training (F _(1, 36)_ = 20.34, *p* < 0.0001) and tone × training interaction (F _(9, 324)_ = 22.54, *p* < 0.0001) but no main effect of treatment (F _(1, 36)_ = 0.39, *p* = 0.54) or any other interactions (not shown). Post-hoc analysis showed that freezing was significantly decreased with immediate, compared to no, extinction at tones 4–10 (*p* < 0.05). This decrease in freezing with immediate extinction later on during extinction recall testing suggests that savings of extinction occurred. The lack of effect of URB597 with no extinction also suggests that fear memory consolidation was unaffected by this drug.

### JZL184 given before immediate extinction enhances fear expression and impairs extinction learning without affecting extinction encoding

The effects of JZL184 (*n* = 10/group) given before immediate extinction on learned fear expression, extinction, and extinction recall are shown in Figure 4. There were no differences in freezing between the groups in response to tone-shock pairings during fear conditioning (Figure 4B). Three-way ANOVA showed no main effects of treatment (F _(1, 36)_ = 0.93, *p* = 0.34) or training (F _(1, 36)_ < 0.001, *p* = 0.99), and no interactions between any of the factors (not shown). There was no difference in freezing before tone presentations during immediate (or no) extinction between the vehicle and JZL184 groups (t _(38)_ = 1.77, *p* = 0.084), indicating that baseline fear expression during immediate extinction was unaffected by JZL184 (Figure 4C). Compared to vehicle, JZL184 increased tone-induced freezing later on during immediate extinction (Figure 4D). Two-way ANOVA showed a marginal main effect of treatment (F _(1, 18)_ = 4.26, *p* = 0.054) and a significant treatment x trial block interaction (F _(4, 72)_ = 4.60, *p* = 0.0023). Post-hoc analysis showed that the increase in freezing with JZL184 reached significance at tone block 4 (*p* < 0.05). There was also significantly lower freezing during tone blocks 2-4 in comparison to the first tone block with vehicle (*p* < 0.05), whereas there were no differences in freezing across the tone blocks with JZL184 (*p* > 0.05). This indicates that JZL184 enhanced cued fear expression and impaired extinction learning. Freezing before tone presentations during extinction recall testing was increased with JZL184, compared to vehicle (Figure 4E). Two-way ANOVA showed a significant main effect of treatment (F _(1, 36)_ = 5.29, *p* = 0.027) but no treatment × training interaction (F _(1, 36)_ = 0.13, *p* = 0.72), indicating that JZL184 resulted in increased baseline fear expression at extinction recall. Tone-induced freezing during extinction recall testing based on mean freezing across the 10 tones was decreased with immediate, compared to no, extinction but this was unaffected by JZL184 (Figure 4F). Two-way ANOVA showed a significant main effect of training (F _(1, 36)_ = 22.30, *p* < 0.0001) but no main effect of treatment (F _(1, 36)_ = 2.89, *p* = 0.098) or treatment × training interaction (F _(1, 36)_ = 0.073, *p* = 0.79). This was confirmed by examining freezing in response to each tone (Figure 4G). Three-way ANOVA showed a significant main effect of training (F _(1, 36)_ = 22.32, *p* < 0.0001) and tone × training interaction (F _(9, 324)_ = 22.70, *p* < 0.0001) but no main effect of treatment (F _(1, 36)_ = 2.90, *p* = 0.097) or any other interactions (not shown). Post-hoc analysis showed that freezing was significantly decreased with immediate, compared to no, extinction at tones 5–10 (*p* < 0.05). This decrease in freezing with immediate extinction later on during extinction recall testing suggests that savings of extinction occurred. The lack of effect of JZL184 with no extinction also suggests that this drug did not affect fear memory consolidation.

### Propranolol given before immediate extinction reduces fear expression without affecting extinction

The effects of propranolol (*n* = 10/group) given before immediate extinction on learned fear expression, extinction, and extinction recall are shown in Figure 5. Freezing in response to tone-shock pairings during fear conditioning did not differ between the groups (Figure 5B). Three-way ANOVA showed no main effects of treatment (F _(1, 36)_ < 0.001, *p* = 0.99) or training (F _(1, 36)_ = 3.03, *p* = 0.090), and no interactions between any of the factors (data not shown). Propranolol decreased freezing before tone presentations during immediate (or no) extinction, compared to vehicle (t _(38)_ = 5.65, *p* < 0.0001), indicating that propranolol reduced baseline fear expression during immediate extinction (Figure 5C). Propranolol also decreased tone-induced freezing during immediate extinction (Figure 5D). Two-way ANOVA showed a significant main effect of treatment (F _(1, 18)_ = 6.41, *p* = 0.021) and treatment x trial block interaction (F _(4, 72)_ = 4.88, *p* = 0.0016). Post-hoc analysis showed that decreased freezing with propranolol reached significance at tone blocks 3–4 (*p* < 0.05), indicating that propranolol decreased cued fear expression during immediate extinction. There were no differences in freezing before tone presentations during extinction recall testing between the vehicle and propranolol groups (Figure 5E). Two-way ANOVA showed no main effect of treatment (F _(1, 36)_ = 0.32, *p* = 0.58) or treatment × training interaction (F _(1, 36)_ = 0.092, *p* = 0.76), indicating that baseline fear expression at extinction recall was unaffected by propranolol. Tone-induced freezing during extinction recall testing based on mean freezing across the 10 tones was increased by propranolol in comparison to vehicle and decreased with immediate, compared to no, extinction (Figure 5F). Two-way ANOVA showed significant main effects of treatment (F _(1, 36)_ = 4.81, *p* = 0.035) and training (F _(1, 36)_ = 8.16, *p* = 0.0071) but no treatment × training interaction (F _(1, 36)_ = 1.41, *p* = 0.24). This effect of propranolol seemed to be driven by increased freezing in the immediate extinction group, which was examined further by assessing freezing in response to each tone (Figure 5G). Three-way ANOVA showed significant main effects of treatment (F _(1, 36)_ = 4.82, *p* = 0.035) and training (F _(1, 36)_ = 8.167, *p* = 0.0071), along with significant tone x training (F _(9, 324)_ = 7.39, *p* < 0.0001) and tone x treatment (F _(9, 324)_ = 2.29, *p* = 0.017) interactions, but there were no other interactions (not shown). Post-hoc analysis showed that freezing was significantly decreased with immediate, compared to no, extinction at tones 7 and 9–10 (*p* < 0.05). However, despite the significant tone × treatment interaction, there were no differences in freezing between the vehicle and propranolol groups at any of the tones (*p* > 0.05). The decrease in freezing with immediate extinction towards the end of extinction recall testing suggests that savings of extinction occurred, while extinction recall did not appear to be affected by prior propranolol treatment. The lack of effect of propranolol with no extinction also suggests that this drug did not affect fear memory consolidation.

## Discussion

In this study we investigated the effects of systemic inhibition of endocannabinoid metabolism on 1) the return of fear over time after delayed extinction, and 2) extinction resistance caused by immediate extinction after recent fear conditioning. We also attempted to replicate previous results demonstrating rescue of the IED by systemic beta-adrenoceptor blockade. In Experiment 1, we found no effects of URB597 or JZL184 given after delayed extinction on extinction recall or the later spontaneous recovery of baseline or cued fear. In Experiment 2, URB597 given before immediate extinction had no acute effect on fear expression or extinction learning. URB597 did result in increased baseline fear expression during extinction recall but cued extinction recall was unaffected. JZL184 given before immediate extinction enhanced cued fear expression and impaired extinction learning acutely. JZL184 also resulted in increased baseline fear expression during extinction recall without affecting cued extinction recall. Propranolol given before immediate extinction reduced baseline and cued fear expression acutely during extinction but had no lasting effect on extinction recall. Moreover, none of these drugs affected fear memory consolidation, given their lack of effect in the no extinction controls. Collectively, these results indicate that endocannabinoid metabolism inhibition had no lasting effects on cued extinction encoding or the return of fear over time, while beta-adrenoceptor blockade failed to rescue the IED. Below we consider various methodological differences between the present study and previous studies on endocannabinoid and adrenergic regulation of fear extinction, which may shed light on the reasons for discrepancies between previous findings and our results.

### Lack of effect of endocannabinoid metabolism inhibition on delayed extinction encoding and later spontaneous fear recovery

Previous studies have shown that FAAH inhibition enhances and MAGL inhibition impairs fear extinction. The FAAH inhibitor AM3506 given before, but not after, extinction facilitated later extinction recall in a mouse strain that shows impaired extinction (Gunduz-Cinar et al., 2013). URB597 given before repeated extinction sessions enhanced extinction recall (Llorente-Berzal et al., 2015). Similarly, URB597 given before the first of three extinction sessions potentiated extinction recall in an inhibitory avoidance paradigm (Segev et al., 2018). URB597 given before two extinction sessions ameliorated the extinction impairment caused by social defeat stress before conditioning (Laricchiuta et al., 2013). Another study showed that URB597 strengthened the extinction of stress-enhanced fear conditioning when given after, but not before, repeated extinction sessions (Morena et al., 2018). In contrast, JZL184 given before repeated extinction sessions impaired extinction learning without affecting its recall (Llorente-Berzal et al., 2015; Hartley et al., 2016; Mizuno et al., 2022). However, in agreement with our results, other studies have also found no effects of FAAH or MAGL inhibition on fear extinction. URB597 given before a single extinction session (Morena et al., 2021) or repeated extinction sessions (Mizuno et al., 2022) had no effect on later extinction recall in males. Similarly, the FAAH inhibitor PF-3845 given before repeated extinction sessions had no effect on extinction encoding (Hartley et al., 2016). Moreover, JZL184 given before or after repeated extinction sessions had no effect on the extinction of stress-enhanced fear conditioning (Morena et al., 2018). The MAGL inhibitor MJN110 given before a single extinction session was also without effect on later extinction recall in males (Morena et al., 2021).

Various methodological differences between previous studies and our study may account for these discrepant findings. It is possible that we may have observed effects with URB597 or JZL184 given before delayed extinction since most studies showing effects of endocannabinoid metabolism inhibition on fear extinction examined the effects of drug treatment before extinction. To our knowledge this is the first study to examine endocannabinoid regulation of fear extinction in Lister hooded rats. This strain was previously shown to be more active and less anxious than other rat strains, which might affect freezing during behavioural testing (Commissaris et al., 2000; McDermott and Kelly, 2008; Clemens et al., 2014). Previous studies have also shown differences in the behavioural effects of direct and indirect CB1R agonists, including URB597, between Lister hooded and other rat strains (Deiana et al., 2007; Seillier et al., 2013; Renard et al., 2013; Basavarajappa et al., 2014; Hlavacova et al., 2015; Hasanein and Teimuri Far, 2015; Matricon et al., 2016; Manduca et al., 2014; Warren et al., 2022b). Chronic CB1R agonism impaired and acute URB597 treatment enhanced novel object recognition in Wistar, but not Lister hooded, rats (Renard et al., 2013; Hlavacova et al., 2015; Hasanein and Teimuri Far, 2015; Warren et al., 2022b), which suggests that the latter strain might be less sensitive to drugs acting on CB1Rs. Previous studies that showed URB597 enhancement of fear extinction used stronger shock parameters during fear conditioning or stress-enhanced fear learning. This suggests that URB597 might preferentially regulate the extinction of stronger fear memory, which is broadly in line with other evidence indicating a role for CB1R signalling in learned fear processing under more aversive conditions in particular (Jacob et al., 2012). We may also have found effects of URB597 with repeated treatments and/or extinction sessions (Laricchiuta et al., 2013; Llorente-Berzal et al., 2015; Morena et al., 2018; Segev et al., 2018).

### Lack of effect of endocannabinoid metabolism inhibition on the encoding of immediate extinction

Although we did not compare directly between delayed and immediate extinction in this study, we did observe a qualitative increase in freezing during the recall of immediate in comparison to delayed extinction. This agrees with our recent results demonstrating the IED by showing impaired extinction recall after immediate, compared to delayed, extinction (Papagianni et al., 2022). Despite using stronger shock parameters during conditioning and giving drug before immediate extinction, we found no effect of URB597 on cued extinction recall. In addition to the methodological issues raised above, studies showing URB597 facilitation of fear extinction used a longer (60–120 min) interval between treatment and behavioural testing than we used here (30 min) (Llorente-Berzal et al., 2015; Segev et al., 2018). This may have contributed to the lack of effect of URB597 on extinction and a limitation of this study is that brain anandamide levels were not quantified. However, other studies have shown behavioural effects of URB597, including enhanced fear extinction, given 30 min before testing (Laricchiuta et al., 2013; Hlavacova et al., 2015; Hasanein and Teimuri Far, 2015; Warren et al., 2022b). In contrast to URB597, we found that JZL184 enhanced cued fear expression during immediate extinction and impaired extinction learning but cued extinction recall was unaffected. These results are in general agreement with previous findings and add to evidence of a role for 2-AG in regulating the expression and short-term extinction of stronger fear memory (Llorente-Berzal et al., 2015; Hartley et al., 2016; Mizuno et al., 2022).

URB597 or JZL184 given before immediate extinction had no effect on cued extinction recall but both resulted in enhanced baseline fear expression during extinction recall testing. Increased baseline fear expression during extinction recall may have resulted from a type of contextual fear conditioning, such that the stress induced by recent conditioning generated a negative interoceptive state that became associated with the extinction context (Bouton et al., 2006; Papagianni et al., 2022). CB1R activation by elevated AEA or 2-AG levels may, therefore, have potentiated this interoceptive state or its contextual association. Although the findings from previous studies on CB1R regulation of contextual fear conditioning are mixed (Pamplona and Takahashi, 2006; Arenos et al., 2006; Butler et al., 2008, 2012; Jacob et al., 2012; Laricchiuta et al., 2013; Hartley et al., 2016; Nasehi et al., 2017; Balogh et al., 2019; Kondev et al., 2022), there is emerging evidence supporting a role for CB1R signalling in regulating negative interoceptive states (Andrade et al., 2019). Another possibility is that URB597 and JZL184 resulted in contextual fear generalization, although there is less support for this idea (Reich et al., 2008).

### Lack of effect of beta-adrenoceptor blockade on the encoding of immediate extinction

To investigate the effects of endocannabinoid metabolism inhibition on immediate extinction we used our recently validated behavioural procedures to induce the IED (Papagianni et al., 2022). Compared to delayed extinction, we used stronger shock parameters with immediate extinction to induce the IED (Maren and Chang, 2006). However, it is worth noting that these shock parameters were weaker than those used by others. These previous studies provided evidence that the IED is mediated at least in part by elevated noradrenaline release resulting from stress caused by recent fear conditioning since propranolol rescued the IED (Fitzgerald et al., 2015; Giustino et al., 2017, 2020). In this study we attempted to replicate this drug effect on the IED using our weaker shock parameters. Although we replicated previous results showing that propranolol dampens baseline and cue-induced fear expression acutely during immediate extinction (Fitzgerald et al., 2015), we found no lasting effect of propranolol on extinction recall. Another recent study also found that propranolol had no effect on the IED but it did ameliorate impaired re-extinction after immediate extinction (Wang et al., 2021). These inconsistent findings on propranolol rescue of the IED may also involve methodological differences between studies. One possibility is that our weaker shock parameters resulted in less involvement of adrenergic signalling, compared to previous IED studies that used stronger shock parameters. Interestingly, evidence indicates that propranolol has the opposite effect on delayed extinction to impair its encoding (Warren et al., 2022a). These opposing effects of propranolol on immediate and delayed extinction are thought to involve different effects of noradrenaline that depend on the state of arousal. Beta-adrenoceptor blockade may allow for the encoding of immediate extinction by reducing the high arousal state resulting from recent fear conditioning, while blocking beta-adrenoceptors during the low arousal state associated with delayed extinction may impair extinction encoding by disrupting optimal noradrenaline transmission (Fitzgerald et al., 2015). Our weaker shock parameters may have resulted in an intermediate arousal state during immediate extinction, which might explain the lack of effect of propranolol on the IED. Other stress mediators, such as corticotropin releasing factor (CRF), have also been shown to play a role in the IED (Hollis et al., 2016; Jo et al., 2020). Therefore, it is possible that the IED induced by our weaker shock parameters may have involved CRF signalling or interactions between CRF and adrenergic signalling, which remain to be examined in future studies.

## Conclusion

We found no effects of endocannabinoid metabolism inhibition on extinction encoding or later spontaneous fear recovery after delayed extinction. Similarly, while MAGL inhibition enhanced fear expression during immediate extinction, neither FAAH nor MAGL inhibition affected its encoding. Our results highlight the importance of various methodological considerations since previous studies have shown that FAAH inhibition facilitates the extinction of stronger fear memories with repeated treatments and/or extinction sessions. From a translational perspective, preferential effects of FAAH inhibition on the extinction of maladaptive fear memory might be desirable in terms of developing FAAH inhibitors as therapeutics to combine with exposure-based therapy to treat anxiety-related disorders. Despite our largely negative results, future studies examining endocannabinoid regulation of fear relapse over time after the extinction of stronger fear memory are therefore still warranted.

## Data Availability

The raw data supporting the conclusion of this article will be made available by the authors, without undue reservation.
